# Monitoring Radiofrequency Ablation Using Ultrasound Envelope Statistics and Shear Wave Elastography in the Periablation Period: An *In Vitro* Feasibility Study

**DOI:** 10.1371/journal.pone.0162488

**Published:** 2016-09-07

**Authors:** Po-Hsiang Tsui, Chiao-Yin Wang, Zhuhuang Zhou, Yung-Liang Wan

**Affiliations:** 1 Department of Medical Imaging and Radiological Sciences, College of Medicine, Chang Gung University, Taoyuan, Taiwan; 2 Medical Imaging Research Center, Institute for Radiological Research, Chang Gung University and Chang Gung Memorial Hospital at Linkou, Taoyuan, Taiwan; 3 Department of Medical Imaging and Intervention, Chang Gung Memorial Hospital at Linkou, Taoyuan, Taiwan; 4 Graduate Institute of Clinical Medical Sciences, College of Medicine, Chang Gung University, Taoyuan, Taiwan; 5 College of Life Science and Bioengineering, Beijing University of Technology, Beijing, China; Taipei Veterans General Hospital, TAIWAN

## Abstract

Radiofrequency ablation (RFA) is a minimally invasive method for treating tumors. Shear wave elastography (SWE) has been widely applied in evaluating tissue stiffness and final ablation size after RFA. However, the usefulness of periablation SWE imaging in assessing RFA remains unclear. Therefore, this study investigated the correlation between periablation SWE imaging and final ablation size. An *in vitro* porcine liver model was used for experimental validation (*n* = 36). During RFA with a power of 50 W, SWE images were collected using a clinical ultrasound system. To evaluate the effects of tissue temperature and gas bubbles during RFA, changes in the ablation temperature were recorded, and image echo patterns were measured using B-mode and ultrasound statistical parametric images. After RFA, the gross pathology of each tissue sample was compared with the region of change in the corresponding periablation SWE image. The experimental results showed that the tissue temperature at the ablation site varied between 70°C and 100°C. Hyperechoic regions and changes were observed in the echo amplitude distribution induced by gas bubbles. Under this condition, the confounding effects (including the temperature increase, tissue stiffness increase, and presence of gas bubbles) resulted in artifacts in the periablation SWE images, and the corresponding region correlated with the estimated final ablation size obtained from the gross pathology (*r* = 0.8). The findings confirm the feasibility of using periablation SWE imaging in assessing RFA.

## Introduction

Radiofrequency ablation (RFA) is a standard alternative treatment in oncologic treatments [1,2]. To guide the RF electrode to the target location of the tumor, surgeons typically use ultrasonography because it provides real-time feedback on the electrode location [3]. RFA-induced coagulation necrosis, which involves protein denaturation and tissue dehydration, increases tissue stiffness [4,5]. Several ultrasound elastography approaches, such as quasistatic elastography [6–10], real-time elastography [11], and acoustic radiation force impulse (ARFI) imaging [12–14], have been explored for RFA monitoring. However, most of these approaches cannot provide quantitative maps for tissue stiffness; therefore, shear wave elastography (SWE) was proposed [15,16].

In SWE, the shear wave velocity is measured to estimate the Young’s modulus of the target tissue. Several previous studies have demonstrated the use of postablation SWE imaging in quantifying the stiffness of RFA-induced lesions *in vitro* and *in vivo* [17–21]. Crucial to ablation assessment is the spatial and temporal stability of tissue stiffness assessment; a recent study explored the use of SWE and ARFI imaging in monitoring the evolution of tissue stiffness at ablation sites during lesion formation in the periablation period [22]. Aside from tissue stiffness, the size of the ablation zone is the other critical factor in physicians’ clinical evaluations of RFA efficiency. However, no previous study has revealed the relationship between periablation SWE and final ablation size.

The present study investigated the correlation between gross examination of the thermal lesion and the region of change in the periablation SWE image. The *in vitro* results indicated that the region of change in the SWE image obtained in the periablation period correlated with the gross pathology. The physical meanings and clinical value of periablation SWE imaging in RFA monitoring are discussed in this paper.

## Materials and Methods

**Fig 1:**
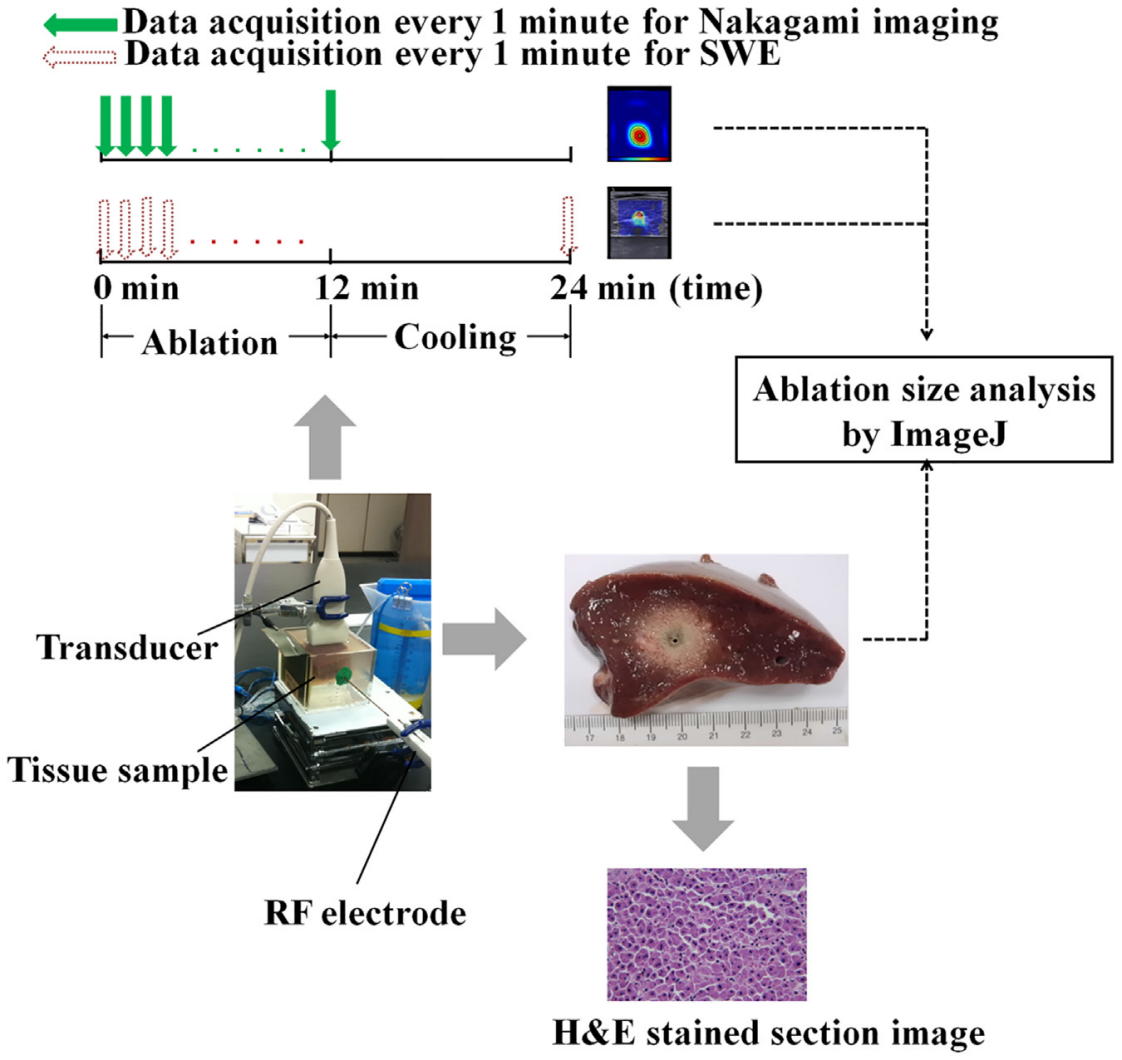
Experimental setting, design, and measurement procedures.

The experimental environment and measurement procedures are illustrated in Fig 1; the details are explained in the following paragraphs.

### In vitro samples

An *in vitro* model based on porcine livers (obtained from the Donshi market at Linkou, Taiwan; WGS84 coordinates: 121.377680, 25.068978) was devised. Each liver sample was cut, placed in a case filled with a saline solution (0.9% NaCl), and compressed in the solution for degassing. Subsequently, each sample was placed in another case filled with a degassed saline solution. A gel phantom was created and applied to the bottom of the case to hold the samples and separate reflection echoes contributed at the bottom from backscattered signals received from the tissue. A metal board was attached to the wall of the case as a grounding pad for the RFA system.

### Thermal lesions

The RFA system (Model VIVA RF generator, Starmed Co. Ltd., Goyang, Gyeonggi, South Korea) employed in the experiments comprised a Cool-tip RF electrode (Model 17-20V15-40, Starmed Co.), an RF generator, a peristaltic pump, cables, and other accessories. The active tip length (ATL) of the electrode can be adjusted to 0.5, 1.0, and 1.5 cm to produce thermal lesions of various sizes. The pump was used to deliver a constant flow of cold saline solution with a mixture of ice to the electrode tip to prevent the electrode from overheating, which would cause tissue carbonization. To measure the tissue temperature corresponding to the location of the electrode tip, a thermocouple was additionally attached to the electrode. Concurrently, the temperature of the electrode tip was automatically recorded by the RFA system. In the experiments, the RF needle electrode was inserted into the liver sample through a small hole created in the case wall. The RFA system was subsequently turned on in clinical default mode (starting power: 50 W; treatment time: 12 min; operation frequency: 480 kHz) and automatically increased by 10 W/min until the RF pulse paused because of high tissue impedance. The system then generated a sequence of RF pulses as a function of time. At the endpoint of ablation (12 min), the RFA system automatically stopped.

### B-mode and SWE imaging

During RFA, periablation B-mode and SWE images of each sample were acquired every minute by using a commercial SWE imaging system (Aixplorer, SuperSonic Imagine, France) equipped with an 8-MHz linear transducer. Postablation B-mode and SWE images were also obtained after the tissue sample was cooled for 24 min for measuring the Young’s modulus by using a built-in circular region of interest (ROI) with a 5-mm diameter centered at the electrode location. All images were collected on a focal plane. For each ATL, independent experiments were conducted on six liver samples.

### Nakagami imaging for bubble detection

Aside from the stiffness increase, RFA also induces gas bubbles in the ablation zone because ablation heating increases the tissue temperature close to the boiling point [7,23]. To examine whether the applied ablation power could induce bubbles during RFA, an additional six liver samples for each ATL (total *n* = 36) were ablated using the same protocol and monitored through ultrasound Nakagami imaging, which is a parametric imaging technique based on the Nakagami parameter of the Nakagami distribution for modeling the echo amplitude distribution [24]. Studies have shown that RFA-induced bubbles significantly change the statistical distribution of backscattered signals, which can be visualized through ultrasound Nakagami imaging [25,26].

The detailed characteristics of the Nakagami imaging system employed in this study has been reported and validated in a previous study [26]. A clinical ultrasound scanner (Model 3000, Terason, Burlington, MA, USA) equipped with a 7.5-MHz linear transducer (Model 10L5, Terason) was connected to a computer; the software employed for real-time Nakagami imaging was developed in the C++ programming language. The algorithmic scheme for Nakagami imaging includes the following steps: (i) conduct frequency diversity by passing the raw image RF signals through two bandpass filters to produce two filtered data, and then compute the corresponding envelope images; (ii) apply the sliding window technique to the two envelope images to obtain their corresponding Nakagami images, which are summed and averaged to yield a compounding Nakagami image; and (iii) apply polynomial approximation to the compounding Nakagami image to visualize the region of change in the echo amplitude distribution (the area within the −6-dB contour was used as the estimate of the bubble region in this study).

### Image analysis

The uses of size or shape parameters for evaluating the ablation zone depend on the type of active electrode. Ablation size is a frequently used parameter when needle-type electrodes are used [19,20,27,28]. The diameter, length, and shape are considered when RFA is performed using an umbrella-shaped electrode [29,30]. Considering the experimental design in the present study, ablation size was used to evaluate the ablation zone.

**Fig 2:**
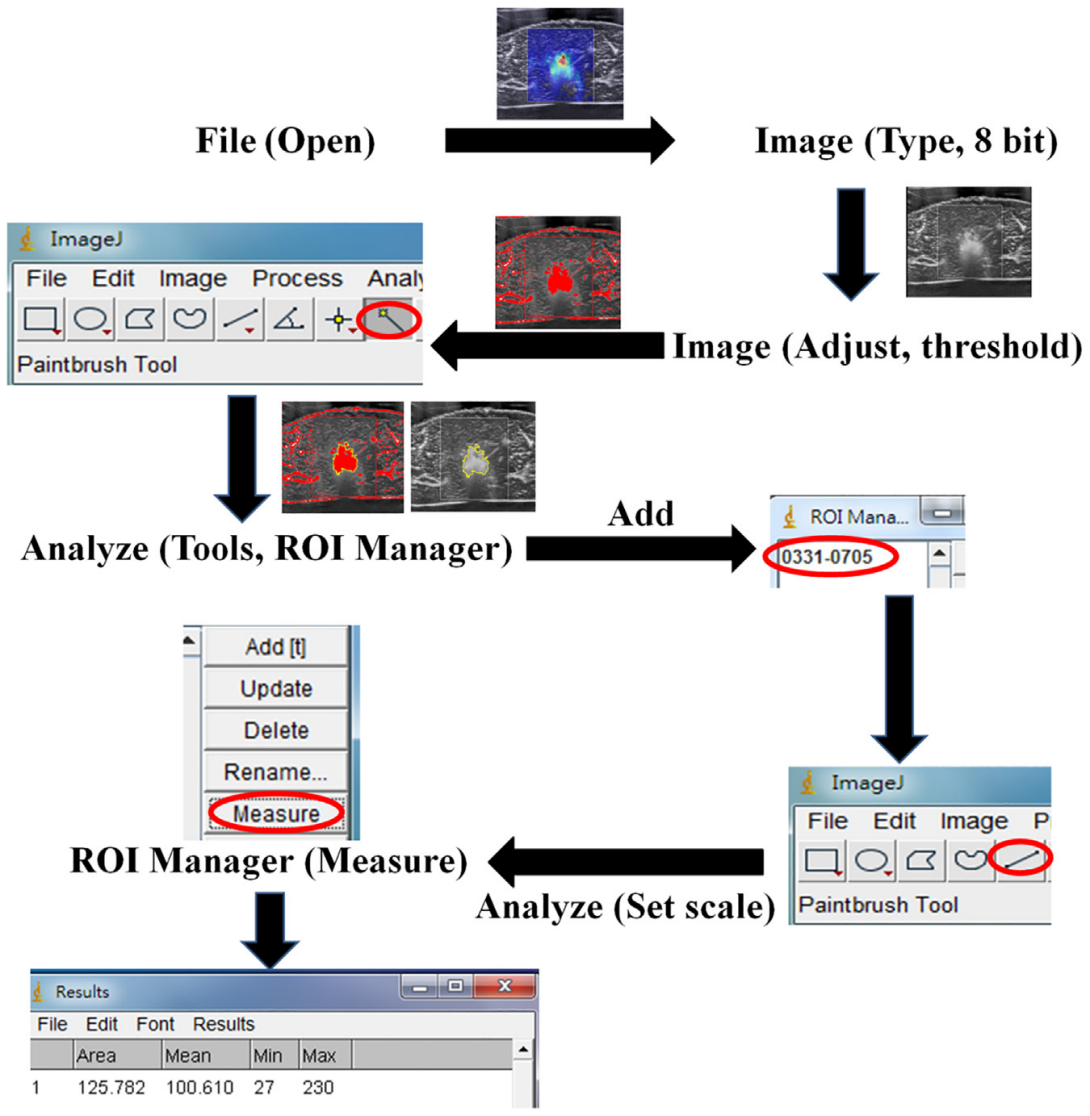
Illustration of the steps for estimating the region of change in the shading of the SWE image through ImageJ. First, the JPEG image file was read and converted into an 8-bit grayscale image. The threshold value—determined by averaging the gray scales in the ROI (approximately 3 × 3 mm) in the unablated zone—was then used to highlight the ablation zone, which was then contoured using the ROI manager in ImageJ to measure the ablation size according to the calibrated pixel scale.

Each periablation SWE image acquired during RFA was analyzed using the software ImageJ (a public domain image processing program developed by the National Institutes of Health) to quantitatively measure the region of change in the shading of the SWE image, denoted by *S*_*SWE*_ with mm^2^ as the unit. The steps for estimating *S*_*SWE*_ by using ImageJ are illustrated and explained in Fig 2. To investigate the behaviors of periablation SWE during RFA, we calculated *S*_*SWE*_ as a function of ablation time. Concurrently, gas bubble generation in the ablation zone is continual during RFA because of heating and the temperature approaching the boiling point. The appearance of gas bubbles can be used to assess the heated region [23], which correlates with the ablated zone [25]. To collect sufficient backscattering information on the bubbles to improve ablation zone visualization, the average of *S*_*SWE*_ acquired at the 1st, 2nd, …, 12th min (i.e., the temporal compounding of images [26]) was also calculated as an overall evaluation of periablation SWE ($\bar{S_{SWE}^{1-12\text{min}}}$). Moreover, the size of the area with changes in the backscattered statistics, denoted by *S*_*Nakagami*_ with mm^2^ as the unit, serving as a function of ablation time, as well as the average of *S*_*Nakagami*_ acquired from the 1st to 12th min ($\bar{S_{Nakagami}^{1-12\text{min}}}$), were calculated to evaluate the behavior of gas bubbles during RFA. The value of *S*_*Nakagami*_ was calculated online through the Nakagami imaging system [26]. Based on the technique of temporal compounding of images, the total measurement time for monitoring one sample to be ablated was determined by the treatment duration of RFA (i.e., 12 min).

### Gross and pathological examinations

After image acquisition, the samples were cut along the ultrasound imaging plane and photographed for gross examination. The photographs (i.e., tissue section images), analyzed using ImageJ, served as the ground truths for measuring the size of the ablation zones. The procedure for processing the tissue section images is shown in Fig 2. To further examine the change in cell morphology caused by RFA, each tissue sample was fixed in 10% neutral buffered formalin, embedded in paraffin, and sliced into 4-μm-thick sections for histological analysis, in which hematoxylin and eosin (H&E) staining was employed. An experienced pathologist determined whether tissue necrosis was induced through RFA.

### Data comparison

The experimental results obtained using different ATLs were compared to calculate the *p* value by using an independent Student’s *t* test. Statistical significance was defined as *p* < 0.05. The measurements obtained from the SWE and Nakagami images were compared with the gross pathology to calculate the correlation coefficient *r* by using a linear curve fitting with the equation form of *y = y*_0_ *+ ax*. Statistical analysis was performed using the software SigmaPlot (Version 9.0, Systat Software, Inc., CA, USA).

## Results

**Fig 3:**
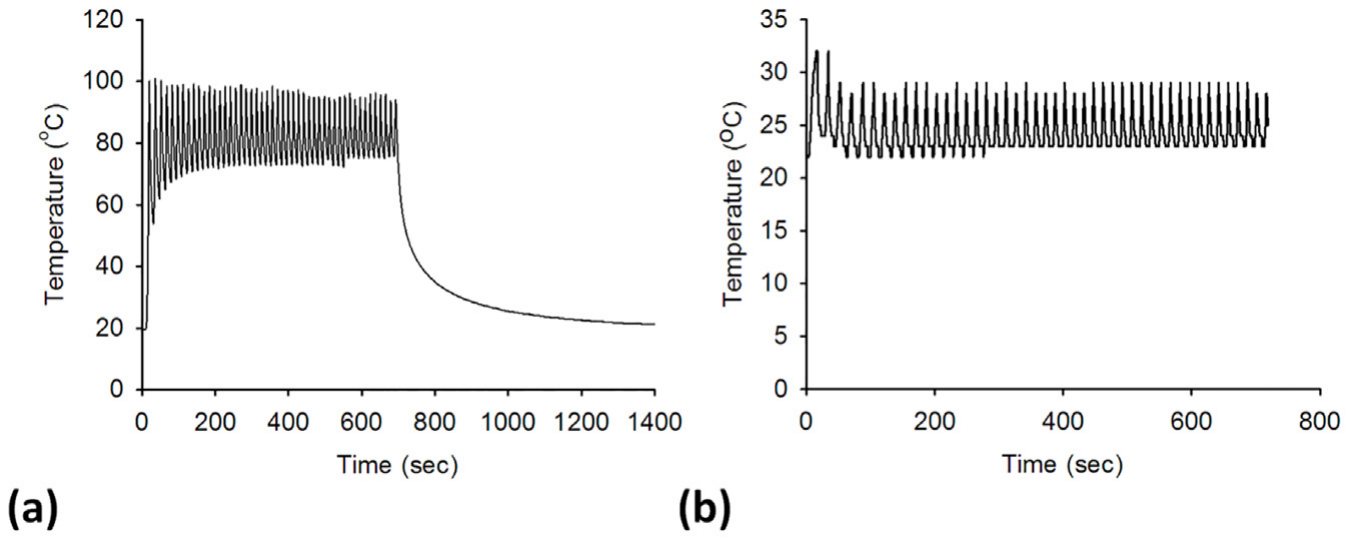
(a) A typical temperature curve as a function of the ablation time, measured using the thermocouple; (b) the temperature curve as a function of the ablation time, recorded from the electrode tip.

**Fig 4:**
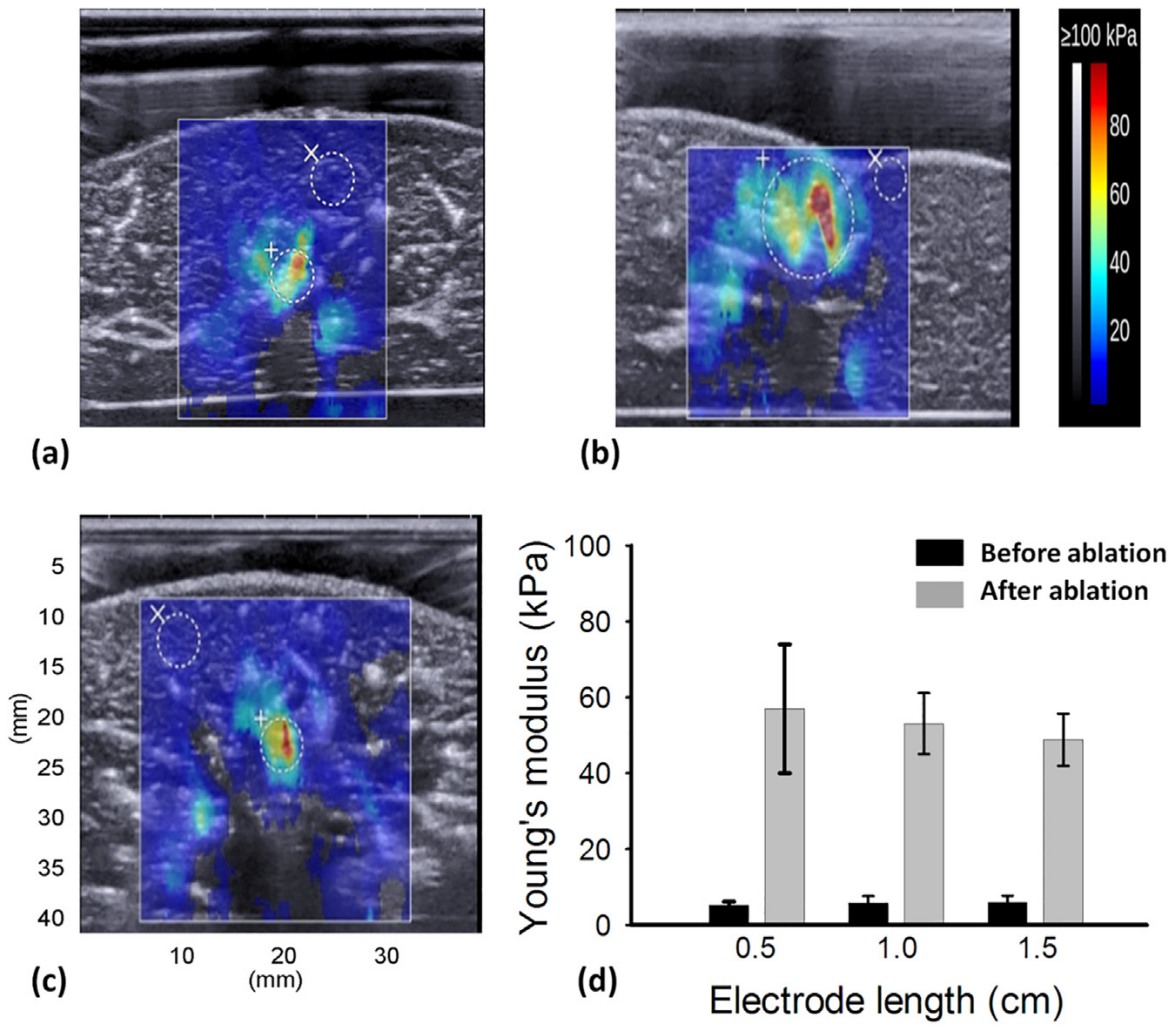
Postablated SWE images of liver samples ablated using ATLs of (a) 0.5 cm, (b) 1.0 cm, and (c) 1.5 cm. (d) Young’s moduli of porcine livers before and after RFA.

Fig 3(a) and 3(b) present typical temperature curves as a function of the ablation time measured from a liver sample and the electrode tip, respectively. During RFA, the tissue temperature varied between 70°C and 100°C. When the RFA system stopped, the tissue temperature rapidly decreased to the initial value. The temperature of the electrode tip varied between 20°C and 30°C during RFA. Fig 4(a)–4(c) show the postablation SWE images of liver samples ablated using ATLs from 0.5 to 1.5 cm. The Young’s moduli of the ablated liver samples varied between approximately 50 and 60 kPa, and were considerably higher than those observed before RFA (*p* < 0.05), as shown in Fig 4(d).

**Fig 5:**
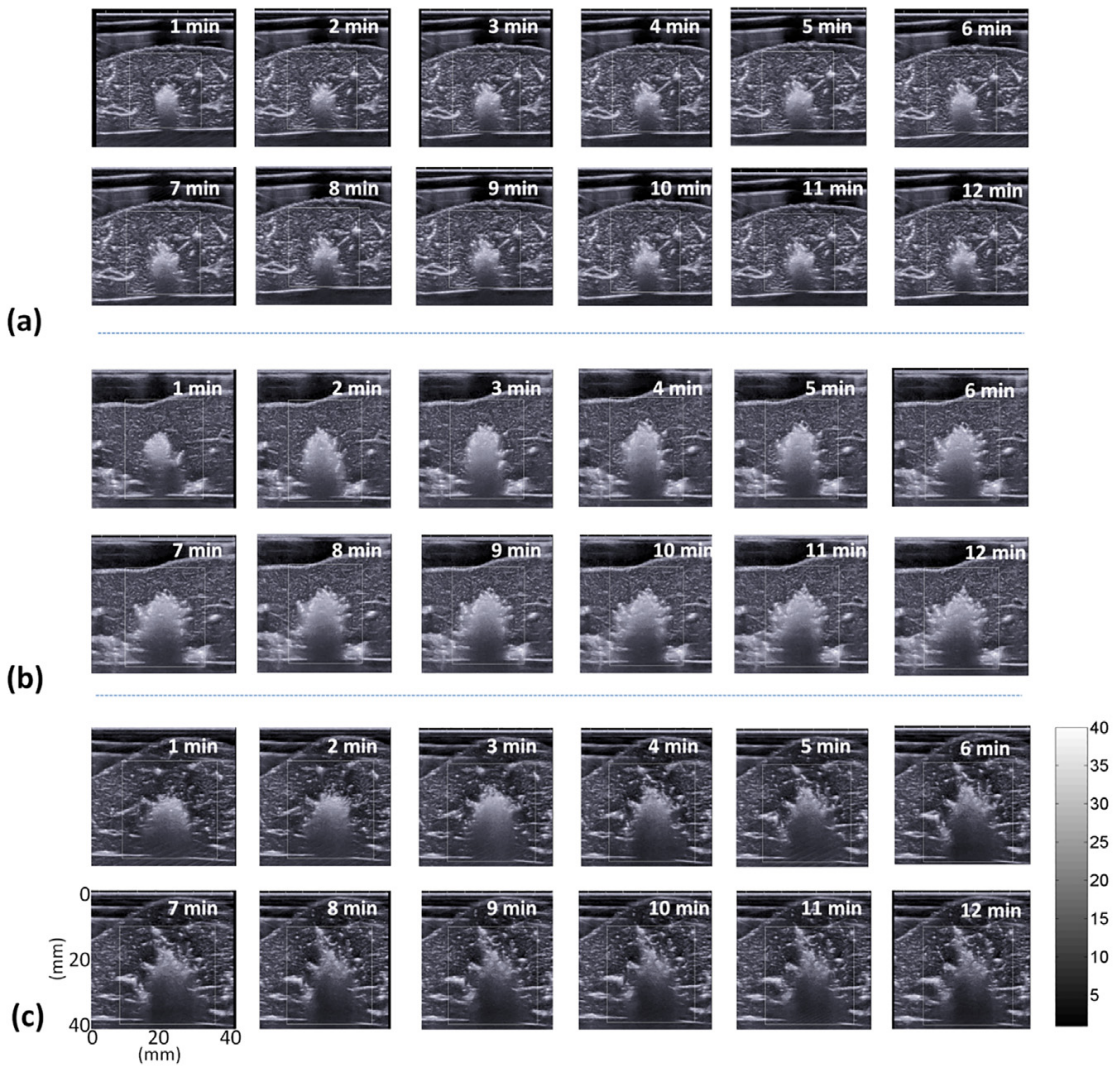
Ultrasound B-mode images acquired at various time points during RFA by using ATLs of (a) 0.5 cm, (b) 1.0 cm, and (c) 1.5 cm.

**Fig 6:**
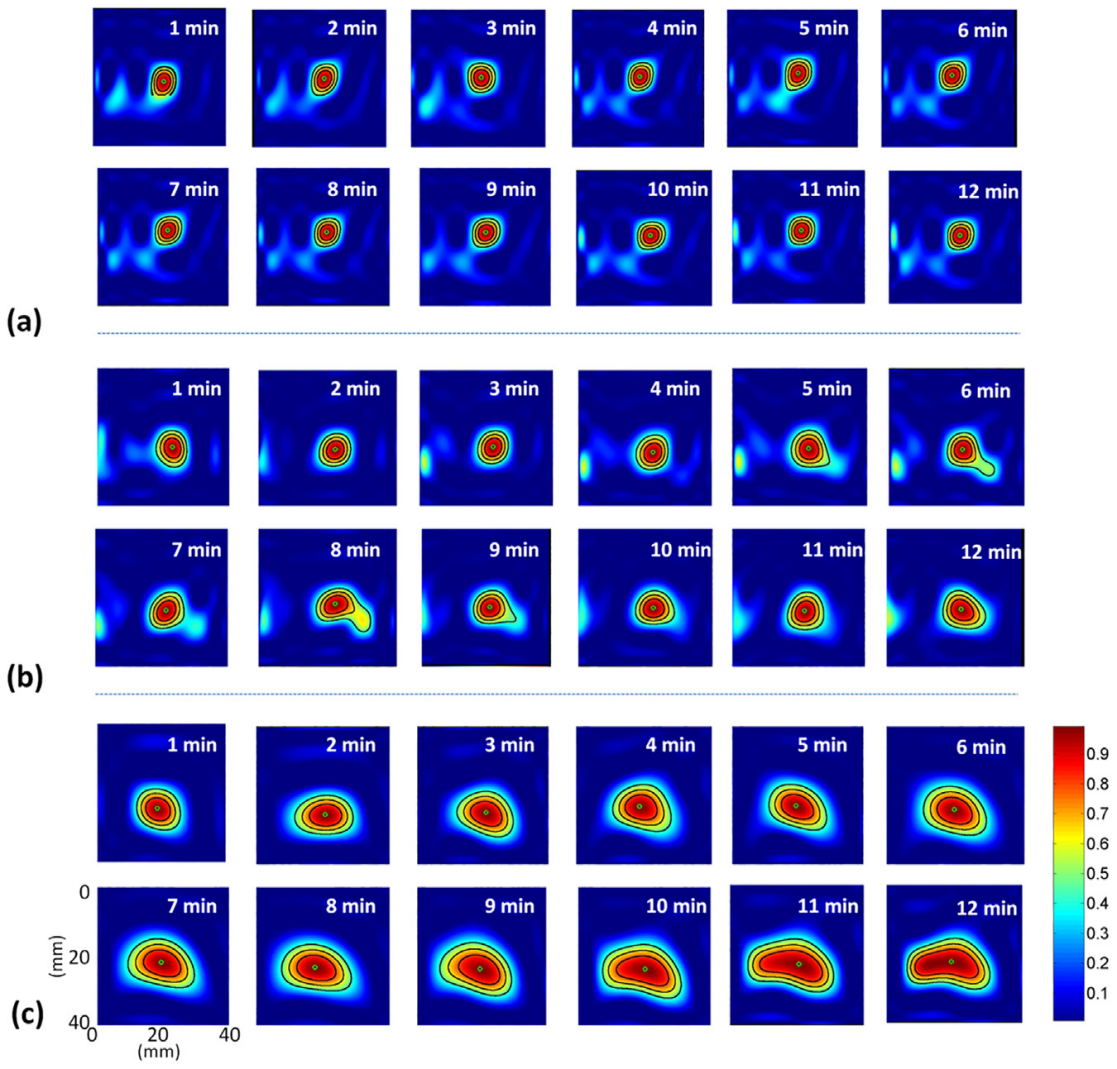
Ultrasound Nakagami images acquired at various time points during RFA by using ATLs of (a) 0.5 cm, (b) 1.0 cm, and (c) 1.5 cm.

**Fig 7:**
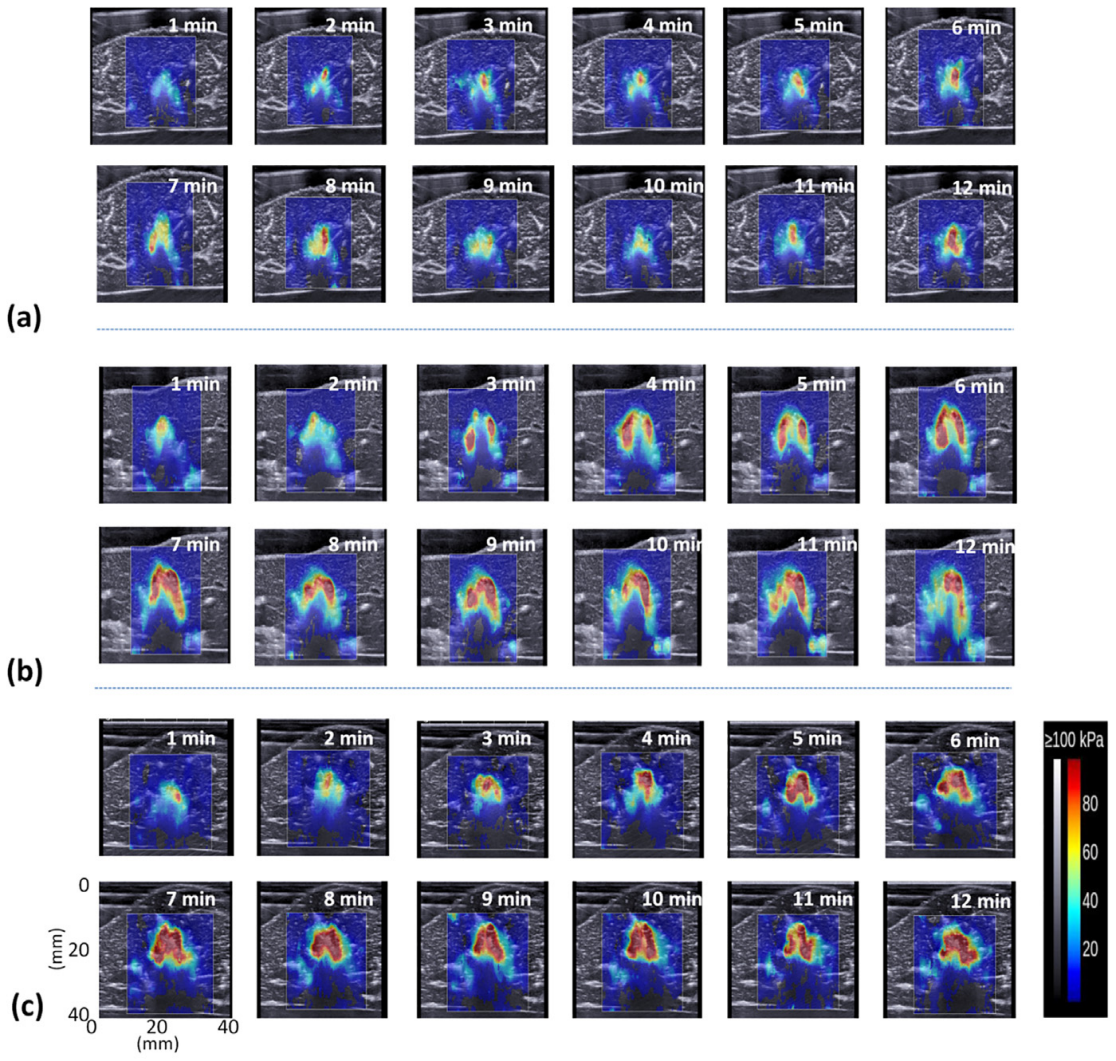
SWE images acquired at various time points during RFA by using ATLs of (a) 0.5 cm, (b) 1.0 cm, and (c) 1.5 cm. These images correspond to the Nakagami images shown in Fig 6.

**Fig 8:**
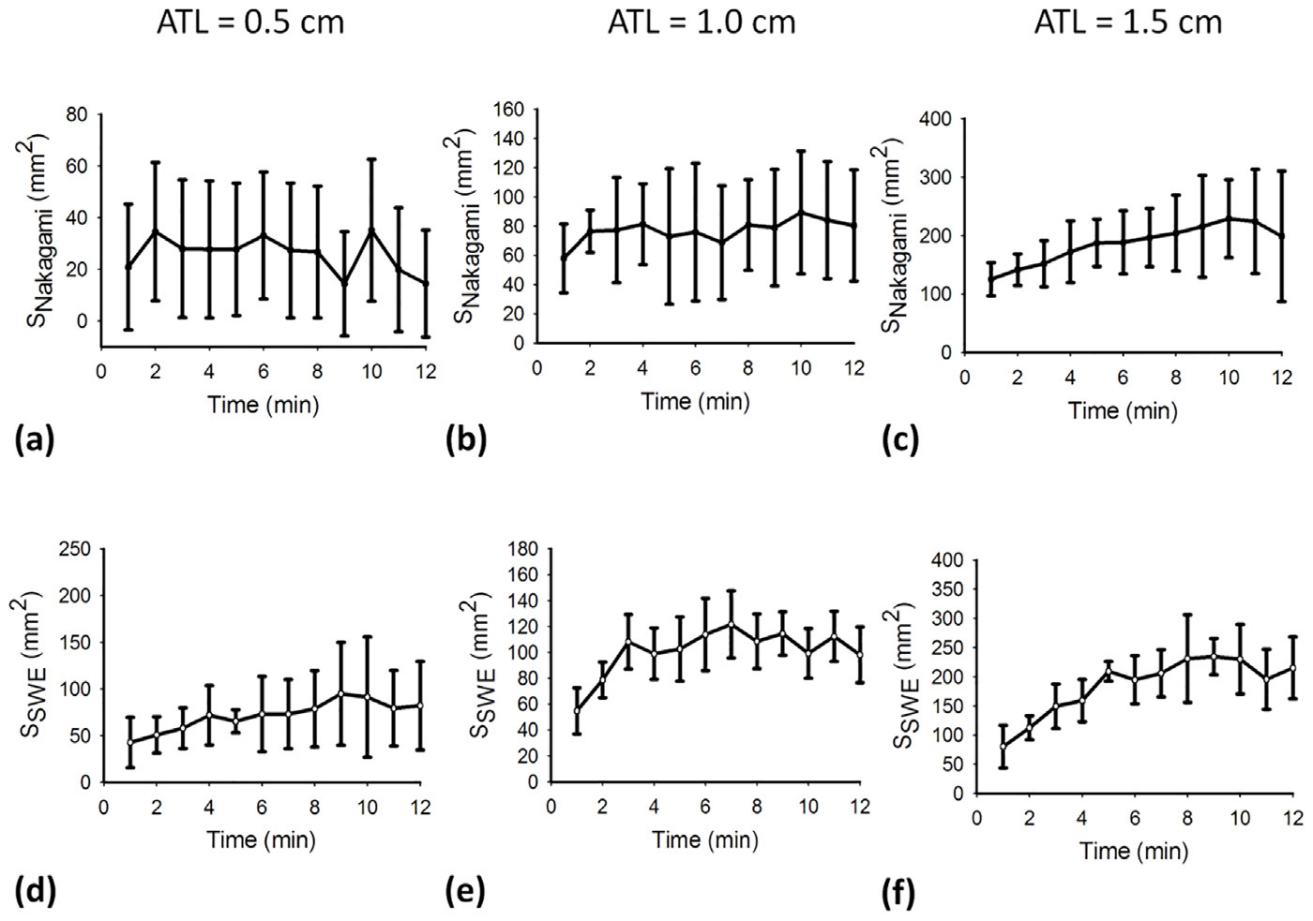
Values of (a)–(c) the size of the area with changes in the backscattered statistics and (d)–(f) the size of the region with changes in the shading of the SWE image as a function of the ablation time, obtained using various ATLs.

**Fig 9:**
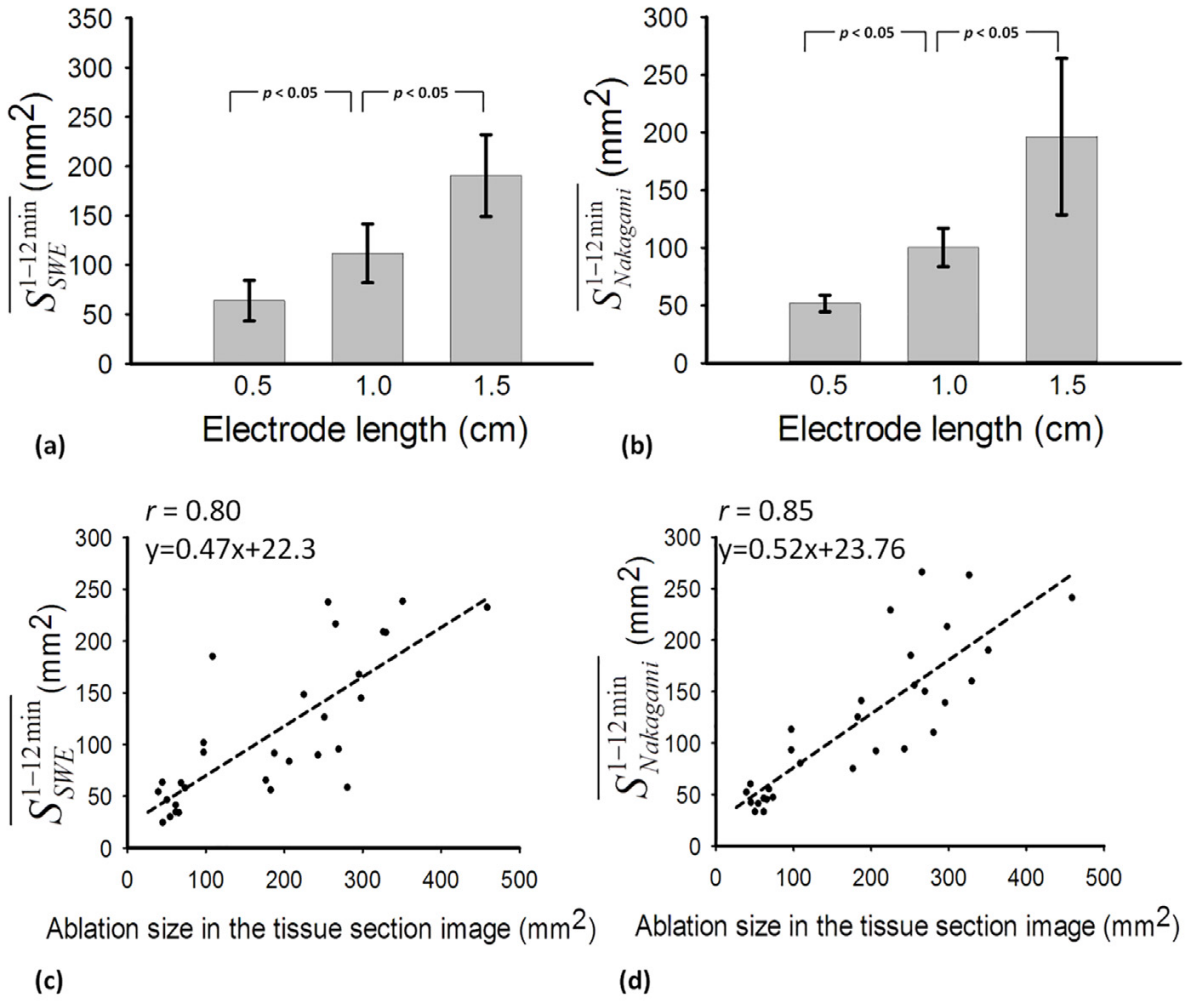
Regions with changes in the shading of (a) SWE and (b) Nakagami images taken during RFA with various ATLs. The ablation sizes obtained from the tissue section images are compared with those obtained from (c) SWE and (d) Nakagami images.

Fig 5 shows the periablation B-mode images obtained using various ATLs. Hyperechoic regions were found in the ablation zone. Regions of change in the backscattered statistics (shown with green to red shading) were also detected in the ablation zone in the periablation Nakagami images, as shown in Fig 6. Periablation SWE images also showed changes in image shading during RFA, as indicated in Fig 7. To indicate the correlation between the gross examinations and visualized regions of change in the periablation images, the results of the quantitative analysis are given in Figs 8 and 9. For each ATL, both *S*_*SWE*_ and *S*_*Nakagami*_ exhibited a slight increase with the ablation time, as shown in Fig 8. The $\bar{S_{SWE}^{1-12\text{min}}}$ value increased from 63.87 ± 20.48 mm^2^ to 190.43 ± 41.45 mm^2^ as the ATL increased from 0.5 to 1.5 cm (*p* < 0.05 between each ATL). In this range of ATLs, the $\bar{S_{Nakagami}^{1-12\text{min}}}$ value increased from 51.75 ± 7.13 mm^2^ to 196.42 ± 67.81 mm^2^ (*p* < 0.05 between each ATL), as shown in Fig 9(a) and 9(b). In particular, Fig 9(c) and 9(d) show that $\bar{S_{SWE}^{1-12\text{min}}}$ and $\bar{S_{Nakagami}^{1-12\text{min}}}$ were closely correlated with the final ablation size (*r* = 0.8 and 0.85 for the SWE and Nakagami images, respectively).

## Discussion

### Research contributions

First, we summarize the significances and findings of this study and then discuss the details later. Note that the relationship between $\bar{S_{Nakagami}^{1-12\text{min}}}$ and the final ablation size was validated in a previous study [26]. However, the dependency of $\bar{S_{SWE}^{1-12\text{min}}}$ on the final ablation size is a new finding. Previously, postablation SWE has been widely explored in evaluating tissue stiffness and ablation size. However, no previous study has reported the usefulness of periablation SWE imaging in estimating ablation size. The present study is the first to conduct *in vitro* experiments to clarify the role of periablation SWE imaging in monitoring RFA. The results show that $\bar{S_{SWE}^{1-12\text{min}}}$ depends on the gross pathology, indicating that using periablation SWE imaging in assessing RFA is feasible.

### Peri- and postablation SWE in stiffness assessment

Clinically successful tumor ablation therapy requires that cells are completely destroyed by substantial thermal effects. The *in vitro* model and experimental setup used in this study provide a simulation of RFA-induced tissue necrosis. As indicated in Fig 3, the tissue temperature at the ablation site varied between 70°C and 100°C. A temperature of approximately 100°C for a 12-min ablation results in a sufficient thermal dose to completely destroy cells.

Tissue necrosis involving protein denaturation and tissue dehydration increases tissue stiffness. Clinically, monitoring the final stiffness of the ablation zone is essential to guarantee the treatment efficacy of RFA and mitigate complications. SWE is currently a state-of-the-art ultrasound tool used for detecting tissue stiffness following RFA. The results in Fig 4 show the use of SWE in stiffness assessment. Previous studies have reported the performance of SWE in monitoring RFA [17–21]. Although different models and RFA systems and parameters have been used in these studies, they have been consistent in their conclusion that final tissue stiffness and ablation size can be effectively assessed using postablation SWE images obtained after tissue cooling. However, under high-power RFA, we noted that the contour of the ablation zone was not clearly visualized in the postablation SWE images, as shown in Fig 4. This may be attributable to the shear wave attenuation. The attenuation of a shear wave in tissues is determined according to the density, shear elasticity, and shear viscosity of the tissue [31]. Thermal ablation increases the shear elasticity and viscosity [32], which continue to increase at temperatures higher than 70°C because tissues lose most of their water content above this temperature [33]. The aforementioned conditions may result in significant shear wave attenuation, which reduces the signal-to-noise ratio of the shear wave signal, thereby influencing the formation of the postablation SWE image and the corresponding image quality.

Regarding the value of periablation SWE images in monitoring RFA, a recent study explored the use of periablation SWE and ARFI imaging for describing changes in tissue stiffness during RFA [22]. That study found that through the SWE and ARFI methods, an immediate increase was detected in tissue stiffness during RFA, suggesting that a consistent stiffness assessment is possible 2 min after and for at least 30 min following ablation. However, the relationship between periablation SWE imaging and the final ablation size was not clarified; furthermore, the physical meanings of the periablation SWE images remain unclear. In our opinion, the physical meanings of periablation SWE imaging only partially correlate with the tissue stiffness because several effects occur during RFA, which are discussed as follows.

### Physical effects and mechanisms of periablation SWE in evaluating ablation size

First, significant thermal effects induced by RFA may affect the biomechanical properties of tissues. For instance, the shear modulus of the liver tissue was nearly constant during heating, increasing exponentially once the tissue temperature exceeded 45°C [34]. In other words, estimation of the tissue stiffness in the periablation period may be affected by the temperature effect. Second, under high-temperature ablation, we observed the image pattern of gas bubbles (i.e., the hyperechoic region) in the B-mode image, as illustrated in Fig 5. Concurrently, a change in the echo amplitude distribution caused by gas bubbles was also detected through Nakagami imaging, as shown in Fig 6. Note that the effects of water vaporization and gas bubble formation during RFA affect SWE imaging [19]. Gas bubbles in aqueous soft tissues may generate a bubble-based radiation force when sonicated by an ultrasound beam [35,36]. The radiation force generated by the bubbles may interact with that of the push beams used for SWE imaging, thereby affecting the formation of the periablation SWE image. Third, to create an SWE image, the speckle motion resulting from shear wave propagation is typically computed using cross-correlation-based techniques on successive images acquired by ultrafast imaging for shear wave velocity estimation. During RFA, new bubbles form, and old bubbles may dissipate (collapse) as the tissue temperature increases [7]. The formation and collapse of bubbles during RFA cause the waveforms of ultrasound signals to vary with time, resulting in computational errors in the cross-correlation analysis between the two acquired images, which results in SWE image artifacts.

According to this discussion, changes in the periablation SWE images obtained during RFA may be attributable to confounding effects including the temperature increase, stiffness (including elasticity and viscosity [32,33]) increase, and bubble-related artifacts. Therefore, the data for periablation SWE images acquired during RFA no longer represent the tissue stiffness. As mentioned, a previous study reported a correlation between the formation of bubbles and the areas being treated by RFA [23]. In this condition, the confounding effects (particularly for bubble-related artifacts) become key factors causing periablation SWE imaging to correlate with the final ablation size, although the confounding effects result in ambiguity of the physical meanings in the periablation SWE images.

### Future work

First, *in vivo* environments were not considered in the employed model. Blood perfusion reduces the ablation temperature, resulting in different ablation volumes, depending on the probe location (e.g., the proximity to a large vessel) [37]; the effect of perfusion on periablation SWE imaging should be further explored clinically. Second, the formation, distribution, and dynamics of gas bubbles occurring during RFA represent complex problems, which result in heterogeneity of periablation SWE images (as shown in Fig 7), and may depend on the properties of the target tissues. The applicability of periablation SWE imaging to various types of tissues is also a crucial research topic. Third, in clinical nodules, the hyperechoic area caused by gas bubbles may disappear and gradually change into the hyperechoic rim around the nodule within 5–15 min after RFA [38]. The hyperechoic rim is related to the necrotic area, although it may be inaccurate [38]. Combining the bubble-related artifacts in periablation images and the hyperechoic rim feature in postablation images may benefit evaluations of clinical RFA.

## Conclusion

In this study, experiments were conducted on an *in vitro* model to explore the correlation between periablation SWE imaging and gross examination of thermal lesions induced by high-temperature RFA. The results reveal that the region of change in SWE images obtained in the periablation period correlates with the gross pathology. Changes in the periablation SWE images may be attributable to the confounding effects based on the temperature increase, stiffness increase, and bubble-related artifacts. The findings of this study confirm the feasibility of using periablation SWE imaging in assessing RFA.

## Supporting Information

S1 Code Source codes for imaging and processing. (RAR)

S1 Data Data for the ablation sizes obtained from SWE, Nakagami, and tissue section images. (RAR)
